# CVTree3 Web Server for Whole-genome-based and Alignment-free Prokaryotic Phylogeny and Taxonomy

**DOI:** 10.1016/j.gpb.2015.08.004

**Published:** 2015-11-10

**Authors:** Guanghong Zuo, Bailin Hao

**Affiliations:** T-Life Research Center, Department of Physics, Fudan University, Shanghai 200433, China

**Keywords:** Composition vector, CVTree, Whole-genome-based tree, Alignment-free phylogeny, Archaea and Bacteria taxonomy

## Abstract

A faithful phylogeny and an objective taxonomy for prokaryotes should agree with each other and ultimately follow the genome data. With the number of sequenced genomes reaching tens of thousands, both tree inference and detailed comparison with taxonomy are great challenges. We now provide one solution in the latest Release 3.0 of the alignment-free and whole-genome-based web server CVTree3. The server resides in a cluster of 64 cores and is equipped with an interactive, collapsible, and expandable tree display. It is capable of comparing the tree branching order with prokaryotic classification at all taxonomic ranks from domains down to species and strains. CVTree3 allows for inquiry by taxon names and trial on lineage modifications. In addition, it reports a summary of monophyletic and non-monophyletic taxa at all ranks as well as produces print-quality subtree figures. After giving an overview of retrospective verification of the **CVTree** approach, the power of the new server is described for the mega-classification of prokaryotes and determination of taxonomic placement of some newly-sequenced genomes. A few discrepancies between **CVTree** and 16S rRNA analyses are also summarized with regard to possible taxonomic revisions. CVTree3 is freely accessible to all users at http://tlife.fudan.edu.cn/cvtree3/ without login requirements.

## Introduction

Prokaryotes are the most abundant and successful organisms on Earth [1]. However, their phylogeny and taxonomic classification had been a long-standing challenge until Carl Woese and coworkers suggested using the small subunit (SSU or 16S) rRNA sequences as molecular markers in the late 1970s [2]. The completion of the second edition of the Bergey’s Manual of Systematic Bacteriology [3] (hereafter referred to as the Manual) marked a culmination of 16S rRNA analysis as the Manual “follows a phylogenetic framework based on analysis of the nucleotide sequence of the small ribosomal subunit RNA, rather than a phenotype structure” (George Garrity’s preface). As early as in 1985, Woese et al [4] proposed a phylogenetic definition for the major eubacterial taxa using all available 16S rRNA sequences, about 400 in total.

The challenge in “congruence” of prokaryotic phylogeny and taxonomy on the basis of SSU rRNA analysis, however, raises a question of principle. In order to establish an objective and valid classification of microbes, the present 16S rRNA-based scheme needs cross-verification. By all means the verification should follow the genomic data. In fact, an Ad Hoc Committee on Reconciliation of Approaches to Bacterial Systematics stated in a 1987 Report that “there was general agreement that the complete DNA sequence would be the reference standard to determine phylogeny and that phylogeny should determine taxonomy” [5]. Since then, genome-based phylogeny and taxonomy studies have been touched on by many research groups from different angles [6], [7], [8], [9], [10].

From a taxonomic perspective, although only a small fraction of the genomes sequenced so far corresponds to prokaryotes with a type strain (1725 among 12,000 [10] as of early 2014), an ambitious program to sequence a myriad of type strains, known as the Genomic Encyclopedia of Bacteria and Archaea project (GEBA) [11], [12], has made rapid progress. The taxonomic coverage of sequenced genomes will soon catch up with that of the 16S rRNA collection.

Only a quarter of a century since the release of Ad Hoc Committee report, with the total number of sequenced microbial genomes reaching tens of thousands, the materialization of “general agreement” has become feasible. However, effectively inferring phylogenetic trees from the genome sequences and comparing the branching orders with taxonomy at all ranks present challenges. To this end, the composition vector (CV) approach to prokaryotic phylogeny developed by our group in the last decade [13], [14], [15], [16], [17], [18], [19], [20], [21], [22], [23], [24] has the potential to meet this challenge. In this paper, we describe the latest version (Version 3) of the CVTree web server and demonstrate its applications. Detailed application of CVTree3 to various aspects of microbiology will be presented in subsequent publications.

## Methods

Since the methodology and foundation of the CVTree approach has been demonstrated in numerous previous publications [13], [14], [15], [16], [17], [18], [19], [20], [21], [22], [23], [24] as well as discussed by other authors [25], [26], we provide only a brief summary of the essentials in this paper.

### CVTree approach

CVTree uses the whole genomes as input, thus avoiding ambiguities in selecting orthologous genes and circumventing the problem of lateral gene transfers. Whole-genome comparison must be alignment-free, as prokaryotic genomes differ significantly in their sizes and gene contents. Our method for conducting alignment-free comparison consists of extending the amino acid alphabet counts to counting the number of K-peptides in all protein products encoded in a genome. In order to highlight the shaping role of natural selection, the original counts are modified by subtracting the random background caused by neutral mutations using a (K–2)-th order Markovian prediction.

In addition to the advantages of using alignment-free data and performing whole-genome-based analyses, several distinctive features of the CVTree are listed below.

1. The peptide length K looks like a parameter but does not function as a parameter because K-values are not adjusted and the same set of K is used for all genomes in a tree. In the older versions of the CVTree server [27], [28], a K-value must be set for each run. CVTree3 carries out calculations for a range of K, such as K = 3–8, in a single run. Watching the branching orders with varying K provides an additional angle for evaluating the quality of the resulting trees. We note that the best (in the sense of agreement with taxonomy) K-values are 4–5 for viruses, 5–6 for prokaryotes, and 6–7 for fungi. Proof of this statement as well as a description of the role of K had been provided in previous reports [16], [23].
2. Traditionally, an inferred phylogenetic tree is subject to statistical re-sampling tests such as bootstrap and jackknife analyses. However, successfully passing these tests only indicates the stability and self-consistency of the tree with respect to small variations of the input data, but not the objective correctness of the phylogeny. Though the CVTree results indeed have passed these time-consuming tests [19], we advocate the viewpoint that phylogenetic trees should be checked directly with taxonomy.
3. The comparison with taxonomy requires a reference classification scheme. In the CVTree server, each built-in genome is associated with initial lineage information taken from the NCBI Taxonomy (www.ncbi.nlm.nih.gov/taxonomy). The information is written in one line with the abbreviations 〈D〉, 〈K〉, 〈P〉, 〈C〉, 〈O〉, 〈F〉, 〈G〉, 〈S〉, and 〈T〉, which stand for Domain, Kingdom, Phylum, Class, Order, Family, Genus, Species, and sTrain, respectively. A standard notation for a lacking classifier is “Unclassified”. For example, “〈F〉Unclassified” denotes a missing family assignment. Lineage information containing one or more “Unclassified” terms is considered incomplete.
4. A central notion in comparing tree branching orders with taxonomy is monophyly. For prokaryotes, while the notion of species is still under debate, one cannot use the original definition of monophyly as the collection of descendants from one and the same common ancestor as discussed by James Farris [29], [30]. Moreover, monophyly is a reciprocal notion with respect to both phylogeny and taxonomy. We adopt a pragmatic approach by restricting ourselves to the input dataset and reference classification. If all genomes from one and the same taxon are represented exclusively by leaves in a single branch, the branch is said to be monophyletic. If a taxon does not appear to be monophyletic in taxonomy, *e.g.*, the genus *Clostridium* consisting of a *sensu stricto* cluster and several “monophyletic” groups as described in volume 3 of the Manual, the corresponding branches cannot be characterized as monophyletic. We use the term “convergence” to describe these cases. A branch may converge to a monophyletic branch such as Cyanobacteria{77} or converge to several partially monophyletic clusters such as *Clostridium*{32/49}, *Clostridium*{7/49}, and *Clostridium*{4/49}, meaning that the 49 genomes listed under the genus *Clostridium* in the reference taxonomy appear as several clusters in CVTree.
5. There are two elements of a phylogenetic tree: the branching order (topology) and the branch lengths. The former reflects taxonomy and the latter is associated with evolution. Calibration of branch lengths is always based on the assumption that the mutation rate has remained constant over evolutionary history, an assumption that cannot hold true when dealing with many phyla in a large-scale study. Although a relationship between the dis-similarity measure used in constructing the CVTree and the usual genetic distance has been derived [16], it does not always preserve the topology of the tree. Therefore, we do not scale branches in all CVTrees and only examine the branching orders. To measure evolutionary time for a group of not-too-distantly-related species, traditional methods such as multi-alignment of orthologous proteins would do the job.

### CVTree3 web server

We have made the CVTree web server publicly available, so bench-biologists can take advantage of the whole-genome-based and alignment-free method. The server has been released twice: Version 1 in 2004 [27] and Version 2 in 2009 [28]. As the CV algorithm is CPU- and memory-demanding, previous servers could not cope with the ever-growing amount of genomic data, and thus we redesigned the CVTree web server. The new CVTree3 server contains many enhanced features and is freely accessible at http://tlife.fudan.edu.cn/cvtree3/. The main improvements are listed as follows. (1) The CV algorithm has been parallelized and the new CVTree3 pipeline now resides in a cluster with 64 cores. (2) The CVTree3 web server is not only designed as a phylogenetic tool, but also enables combined study of phylogeny and taxonomy both on a large scale across many phyla and at lower ranks down to infrasubspecific strains. (3) The server is equipped with an interactive tree display, allowing for the collapse and expansion of branches in accordance with lineage information associated with the input genomes. (4) The server reports the number of genomes in all monophyletic and non-monophyletic taxa/branches at all ranks from the domain down to the species. (5) The server allows for trial lineage modifications and re-collapsing of the tree with a new report on monophyly. (6) The server allows print-quality output of any selected subtree.

Since there is a detailed online (and printable) User’s Manual (File S1) for the web server, we will not describe in detail the aforementioned technical points except to demonstrate some useful features regarding the taxonomic placement of a few newly-sequenced genomes without proper lineage information given at the present time.

### Genome resources

Inherited from the previous releases, the CVTree3 web server has a built-in genome dataset. However, because the NCBI FTP site (ftp://ftp.ncbi.nih.gov/genomes/Bacteria/) has nearly stopped releasing new bacterial genomes since the beginning of 2014, CVTree3 has to give up monthly automatic updating from the NCBI. Currently, prokaryotic genomes are collected from the NCBI, European Nucleotide Archive (ENA) at the EBI, Integrated Microbial Genomes (IMG) by the Joint Genome Institute (JGI) of the U.S. Department of Energy (DOE), the Broad Institute, the J. Craig Venter Institute, and the Pathosystems Resource Integration Center (PATRIC) (the URLs of these institutions are listed in CVTree3 User’s Manual, see File S1). In order to demonstrate the capability of the CVTree, we also included some genomes from the Microbial Dark Matter Project [31].

In this article, we refer to a fully-fledged Working Project to demonstrate the features of the CVTree3 web server. The project has a specific project number 30150127_1559_28802 in order to avoid being deleted by the web server in due time. In the input dataset, there are 342 *Archaea*, 2870 *Bacteria*, and 8 *Eukarya* genomes. The latter includes 4 fungal and 4 non-fungal genomes serving as candidates of the out-group in tree construction.

Users may upload their own genomes together with lineage information. In the Working Project, a total of 21 genomes were uploaded and their names appeared in the “Upload User Genomes and Lineage File” page. The user-supplied lineage information file carries a fixed name “Lineage.txt” and it does not appear explicitly on the page.

The CVTree approach depends on genome annotation, but is insensitive to the annotation mainly because of the alignment-free methodology. For example, genomes of *Gluconacetobacter diazotrophicus* PAI 5 from the same source (ATCC 49037) were sequenced, assembled, and annotated by two institutions. These assemblies contained “a surprisingly high number of differences” [32], yet they appeared in CVTrees as two closely-related sisters. In addition, two genomes listed under an unclassified bacterial phylum *Acetothermia* from IMG JGI provided another example of the insensitivity of CVTree to annotation. Both genomes are in the permanent draft status. GenBank files may be generated from contigs using the IMG pipeline. Their names appeared in the User Uploaded Genomes in our Working Project (Acetothermia bacterium SCGC AAA255 C06 SAK 001 122 and *Candidatus* Acetothermum autotrophicum). By using the “Search Query” function of the interactive display, the genomes appear together in CVTree at the phylum level. The fact that CVTree can accept some permanent-draft genomes greatly widens the reach of CVTree, as there are more than 23,000 permanent drafts according to the Genomes Online Database (GOLD, gold.jgi-psf.org) statistics, and this number is increasing rapidly.

### Applications of CVTree3

Given an input genome set and a parameter-free method such as CVTree, an inferred tree is a fixed unchangeable subject, and the tree cannot be adjusted or modified. In contrast, taxonomy has always been a work in progress. Lineage modifications and taxonomic revisions are routine issues, leading to a convergent phylogeny-based classification of microbial organisms. Over the years, CVTree has been applied to viruses [33], [34], *Archaea* and *Bacteria* [13], [14], [15], [16], [17], [18], [20], [21], [22], [24], chloroplasts [35], and fungi [36] with remarkable success. The powerful and parallelized CVTree3 web server will bring about many additional new applications.

**Figure 1:**
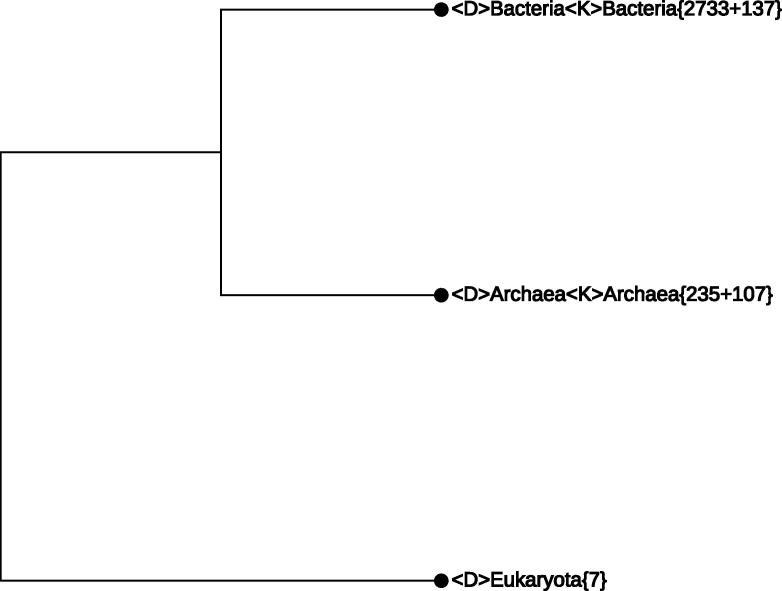
**The most collapsed CVTree with three main domains of life** Note that all 3219 genomes are visible in this single screen (a eukaryotic genome used as the outgroup was hidden). The {n + m} notation indicates that there are n genomes with complete lineage information and m genomes with incomplete or missing lineage information. {n + m} is indicated as {n} when m = 0, while when n = 0, {n + m} is indicated as {0 + m}. 〈D〉 and 〈K〉 represent domain and kingdom, respectively. Main domains of life were defined as suggested by Woese and Fox [37]. “Unclassified” indicates missing classifier. Lineage information containing one or more “Unclassified” is considered incomplete.

Upon entering the Working Project, a maximally-collapsed CVTree with three branches, corresponding to the three main domains of life, appears as shown in Figure 1. All 3220 genomes are represented in this single screen. Bacteria{2733 + 137} indicates that there are 137 bacterial genomes without proper lineage information. A complete lineage may also require modification in order to reflect the actual taxonomic position. By introducing lineage modifications, these numbers may change, but their sum remains at 2870. By expanding the nodes or making enquiry for a designated taxon name, any part of the tree may be unfolded for in-depth inspection.

### Retrospective verifications of CVTree

Before describing the applications of CVTree3, we recall the significant fact that for prokaryotes with sequenced genomes, all taxonomic revisions or new proposals published thus far agree with CVTree or at least do not contradict the CVTree branching order. These should be regarded as retrospective verifications of the new approach. A partial list follows.

1. The move of the genus *Oceanobacillus* from the phylum *Proteobacteria* [38] to phylum *Firmicutes* in 2003 [39].
2. The move of the species *Thiomicrospira denitrificans* from the class *Gammaproteobacteria* to the class *Epsilonproteobacteria* as *Sulfurimonas denitrificans* in 2004 [40], with the reclassification proposal published in 2006 [41].
3. The reassignment of *Thermoanaerobacter tengcongensis* to a new genus as *Caldanaerobacter tengcongensis* in 2004 [42].
4. The transfer of *Thermomicrobium roseum* from its original phylum *Thermomicrobia* to class *Thermomicrobia* in the phylum *Chloroflexi* in 2004 [43].
5. The reclassification of *Sphaerobacter thermophilus* from the phylum *Actinobacteria* to the class *Thermomicrobia* in phylum *Chloroflexi* in 2004 [43].
6. The transfer of *Enterobacter sakazakii* to a newly-proposed genus as *Cronobacter sakazakii* in 2008 [44] led to a monophyletic *Cronobacter*{6} in the current CVTree.
7. The reclassification of a few *Clostridium* and *Ruminococcus* species to a newly-proposed genus *Blautia* in 2008 [45] led to a monophyletic *Blautia*{6} in current CVTree.
8. The suggestion to exclude *Actinobacillus succinogenes* and ‘*Mannheimia succiniciproducens*’ from their respective genera in 2008 [46] and the proposal to establish a new genus *Basfia* to accommodate similar succinic acid-producing bacteria in 2010 [47] led to three monophyletic genera *Mannheimia*{8}, *Actinobacillus*{4}, and *Basfia*{2} in CVTrees.
9. The class *Mollicutes* never joined the other two classes *Bacilli* and *Clostridia* of the phylum *Firmicutes* since the first CVTree was published in 2004. It was removed from volume 3 (2009) of the Manual on *Firmicutes* to become a new phylum *Tenericutes* in volume 4 of the Manual in 2010.
10. The reclassification of *Bacillus tusciae* to a new genus as *Kyrpidia tusciae* in the family *Alicyclobacillus* in 2011 [48].
11. The assignment of *Thermobaculum terrenum* to the phylum *Chloroflexi* in 2011 [49].
12. The move of *Clostridium difficile* and other clostridial species to new genera such as *Peptoclostridium*, *Lachnoclostridium*, and *Ruminoclostridium* in 2013 [50], leading to a monophyletic *Peptoclostridium*{12} in the current CVTree. We note that these names are effectively published but have not been validly published, based on their absence in LPSN [51].
13. The reclassification of *Agromonas oligotrophica* into *Bradyrhizobium oligotrophicum* in 2013 [52] led to a monophyletic *Bradyrhizobium*{6} in the current CVTree.
14. The reclassification of *Thermoproteus neutrophilus* to *Pyrobaculum neutrophilum* in 2013 [53] led to two monophyletic genera *Thermoproteus*{2} and *Pyrobaculum*{8} in CVTree.
15. A recent proposal to elevate four families in the class *Actinobacteria* to corresponding single-family orders [54] does not contradict the current CVTree. In particular, the accommodation of the three genera *Geodermatophilus*, *Blastococcus*, and *Modestobacter* in the order *Geodermatophilales* is supported by CVTree.
16. A recent proposal to split the euryarchaeal order *Halobacteria* into three orders [55] is supported by CVTree [24].

To describe the possible applications of CVTree3 to microbiology, we chose a few topics to demonstrate this potential rather than to explore biological details. These include the following: large-scale classification, taxonomic placement of newly-sequenced genomes, and high resolution of CVTree at the rank species and below.

### Mega-classification of prokaryotes

Large-scale classification, or as Cavalier-Smith puts it [56], mega-classification, of prokaryotes, deals with higher taxonomic ranks such as phylum, class, and order (at present, ranks higher than order are not covered by the International Bacterial Code [57]). The second edition of Bergey’s Manual [3] lists 2 archaeal and 26 bacterial phyla. The total number of prokaryotic phyla may be in the hundreds. In fact, some newly-sequenced genomes represent yet unclassified phyla or classes. The Working Project accompanying this paper helps to comprehend the overall situation. Since a comparison of the archaeal phyla has recently published [24], we concentrate on bacterial taxa.

If the “Modified Lineage” box in the setting-parameter page is checked, a default Lineage Modification file is used to report the convergence of taxa. If unchecked, the initial information from the NCBI Taxonomy is used. In this “bare” situation, an overwhelming majority of phyla appear to be well-defined, *i.e.*, monophyletic for at least one K-value and occupying a position at the phylum level. These phyla include *Acidobacteria*{7 + 2}, *Actinobacteria*{365 + 2}, *Aquificae*{14}, *Chlamydiae*{141}, *Chlorobi*{13}, *Cyanobacteria*{2 + 75}, *Deferribacteres*{4}, *Deinococcus-Thermus*{20}, *Dictyoglomi*{2}, *Fibrobacteres*{3}, *Fusobacteria*{8}, *Planctomycetes*{7}, *Synergistetes*{5}, *Thermotogae*{19}, and *Verrucomicrobia*{2 + 2}. In particular, we point at the relatively-unresolved *Cyanobacteria* phylogeny{2 + 75}. The large number (75) of incomplete lineage information reveals the long-due challenge of *Cyanobacteria* taxonomy. Historically, classification of Cyanobacteria followed the Botanic Code. Currently, NCBI Taxonomy and the Bergey’s Manual differ significantly for *Cyanobacteria*. The complete solution of this issue should be examined further.

Some “big” phyla, *i.e.*, those represented by a large number of genomes, naturally appeared to be non-monophyletic when no lineage modification was made. These include *Bacteroidetes*{88/89 + 5}, *Firmicutes*{662}, *Proteobacteria*{1175}, and *Spirochaetes*{59}. We also encounter a few interesting cases, in which the CVTree results differ from 16S rRNA-based taxonomy. For example, until recently, the species *Thermodesulfovibrio yellowstonii* had been considered a member of the phylum *Nitrospirae* [58]. However, in CVTree, it is resolved to the phylum *Thermodesulfobacteria*. This lineage modification according to CVTree would lead to monophyletic resolving of *Nitrospirae*{4} and *Thermodesulfobacteria*{3}. Similarly, the family *Rhodothermaceae* with its two subordinate genera *Salinibacter* and *Rhodothermus* belongs to an uncertain order (*Bacteriales* Order II *Incertae Sedis* in the Manual) of the phylum *Bacteroidetes*. But in CVTree, it is the nearest neighbor to the phylum *Chlorobi*. Corresponding lineage modification leads to monophyletic *Bacteroidetes*{88 + 5} and *Chlorobi*{13 + 5}.

The clearest distinction of the CVTree phylogeny and 16S rRNA analyses arises from the phylum *Spirochaetes*. Species in this phylum were placed together mainly based on their morphological similarities in the first edition of the Manual. Carl Woese described that spirochaetes form a single clade according to 16S rRNA features [4]. Taxonomically, the phylum *Spirochaetes* consists of a single class, which in turn is composed of one order. Therefore, only the rank family makes sense. In CVTree, the three monophyletic families *Spirochaetaceae*{44}, *Brachyspiraceae*{7}, and *Leptospiraceae*{8} do not join each other, but the first two are closer together.

Three out of five classes in the phylum *Proteobacteria*{1175} appear to be monophyletic clusters, including *Alphaproteobacteria*{257 + 9}, *Betaproteobacteria*{150 + 14}, and *Epsilonproteobacteria*{104 + 3}. Taking into account that the *Beta*- and *Gamma*- groups together form a greater monophyletic cluster, only the class *Deltaproteobacteria*{61} challenges the present classification. There is a core 〈C〉*Deltaproteobacteria*{43/61}, an order 〈O〉*Myxococcales*{13} in the neighborhood of 〈P〉*Acidobacteria*, an order 〈O〉*Bdellovibrionales*{4} joining 〈F〉*Leptospiraceae* as a sister group, and an outlier 〈G〉*Hippea* escaping to 〈P〉*Aquificae*. In a sense, only the phylum *Firmicutes*{662} awaits essential taxonomic revision. Historically, many phyla have been taken out from *Firmicutes*, *e.g.*, *Actinobacteria* and *Tenericutes*. The taxonomy of some genera such as *Clostridium* remains unsettled, although *Clostridium* has been modified after separating five genera in 1994 [59] and six genera in 2013 [50]. CVTree3 may contribute to the further resolution of this problem.

### Taxonomic placement of newly-sequenced genomes

**Figure 2:**
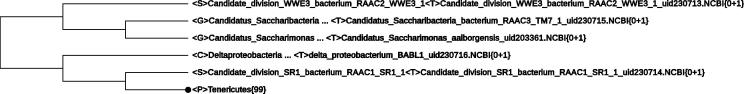
**Candidate taxa at the phylum level near *Tenericutes*** The CVTree3 server collapses candidate taxa at the phylum level near *Tenericutes* to a single note 〈P〉*Tenericutes*{99 + 5}. The {n + m} notation indicates that there are n genomes with complete lineage information and m genomes with incomplete or missing lineage information. {n + m} is indicated as {n} when m = 0, while when n = 0, {n + m} is indicated as {0 + m}. 〈P〉, 〈C〉, 〈G〉, 〈S〉, and 〈T〉 stand for phylum, class, genus, species, and strain, respectively. “Unclassified” indicates missing classifier. Lineage information containing one or more “Unclassified” is considered incomplete.

The number of unclassified phyla far exceeds that of known phyla. On the SILVA web page for candidate taxonomic units (ftp.arb-silva.de/release_108/), 424 “phyla” are numbered among one of the 15 groups in the OD1 group only. Fortunately, owing to the advent of relatively inexpensive and effective sequencing technology, these phyla are beginning to be discovered. Currently, genome-based phylogeny provides the only means for judging the taxonomic placement of genomes without phenotyping data. In CVTree, the collapsing mechanism helps group together closely-related genomes at the phylum level and above. Although not an exhaustive list, we indicate that (1) *Caldiserica* and *Coprothermobacter* (listed under *Firmicutes* in the Manual but considered as an “established phylum” in a 2004 microbial census [60]) are within the branch of 〈P〉*Thermotogae*{19} and 〈P〉*Dictyoglomi*{2}; and (2) Candidate division WWE3, *Candidatus* Saccharibacteria, and *Candidatus* Saccharimonas are located next to 〈P〉 *Tenericutes*{99 + 2}. Further expansion of the last taxon reveals {+2} to be a member of the candidate division SR1 and a misclassified delta-proteobacterium BABL1. Their relationship is shown in Figure 2.

Details of similar cases are not provided due to limited space in this paper. Interested readers are recommended to consult the example Lineage Modification file (File S2) for plausible lineage modifications.

### On CVTree “outliers” as compared with 16S rRNA taxonomy

Every classification depends on characters and criteria used. There is no *a priori* reason that 16S rRNA analysis and whole-genome approaches should yield identical results. The fact that they agree with each other in an overwhelming majority of cases confirms the objectivity of the present 16S rRNA-based taxonomy. However, minor discrepancies cannot be ignored, and these differences should be recorded and further studied. In addition to the aforementioned cases such as the phylum *Spirochaetes*, the class *Deltaproteobacteria*, the orders *Myxococcales* and *Bdellovibrionales*, and the “genus” *Coprothermobacter*, we also include a few more as follows. (1) A new lineage from the genus *Dehalococcoides* to the class *Dehalococcoidia* was proposed recently within the phylum *Chloroflexi* [61]. However, the 9 genomes from this taxon in CVTree, though forming a stable cluster, are not part of *Chloroflexi* and probably comprise a separate phylum. (2) *Magnetococcus marinus* was recently proposed to be a single-species lineage from genus to order at the base of the class *Alphaproteobacteria* [62]. Although this species surely belongs to the phylum *Proteobacteri*a, it was separated from the main body of *Alphaproteobacteria* by a group of insect symbionts with highly-degenerated genomes. Whether this phenomenon is an artifact caused by the influence of their very small genomes [9], [16] requires further analysis. (3) *Hippea maritime* definitely escapes from *Deltaproteobacteria* to the neighboring phylum *Aquificae*.

**Figure 3:**
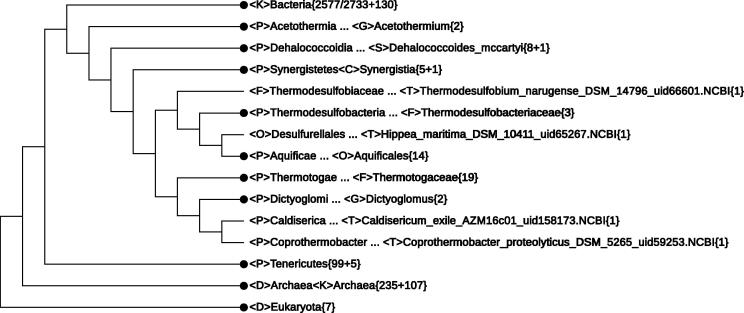
**A 3220-genome CVTree collapsed to highlight the position of *Acetothermia*, *Dehalococcoidia*, *Hippea*, and *Coprothermobacter*** The {n + m} notation indicates that there are n genomes with complete lineage information and m genomes with incomplete or missing lineage information.〈D〉 and 〈K〉, 〈P〉, 〈C〉, 〈O〉, 〈F〉, 〈G〉, 〈S〉, and 〈T〉 stand for domain, kingdom, phylum, class, order, family, genus, species, and strain, respectively. “Unclassified” indicates missing classifier. Lineage information containing one or more “Unclassified” is considered incomplete. The fraction 2577/2733 means 2577 from a total of 2733 genomes.

Figure 3 is a tree based on all 3220 genomes, with the region of interest expanded to the rank of phylum and the rest collapsed as much as possible. Most points discussed above can be observed in this figure. Note that summing up the number of genomes shown explicitly in this figure yields 3219, because one genome used as out-group was hidden.

### Infrasubspecific interrelationship within species

A prominent feature of CVTree is its high resolution at the species level and below (for infrasubspecific ranks, see page 30, volume 2 of the Manual), far surpassing the capability of 16S rRNA analysis. Moreover, the simplicity of obtaining a subtree image is remarkable. Once the genomes are submitted to the CVTree web server, fine branching for all species is produced in one run. There is no need to collect orthologous proteins and to conduct multiple alignments. We provide a few examples to show that the resulting subtrees make sense.

**Figure 4:**
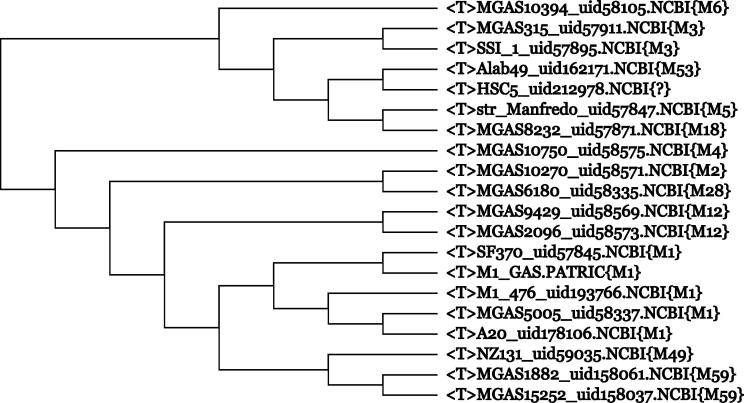
**Serotypes of the *Streptococcus pyogenes* strains** Only strain tags are shown. Serotypes are given at the end of each entry. Whether the unknown {?} is M53 may be tested when serotype of the strain becomes available. 〈T〉 stands for strain.

**Figure 5:**
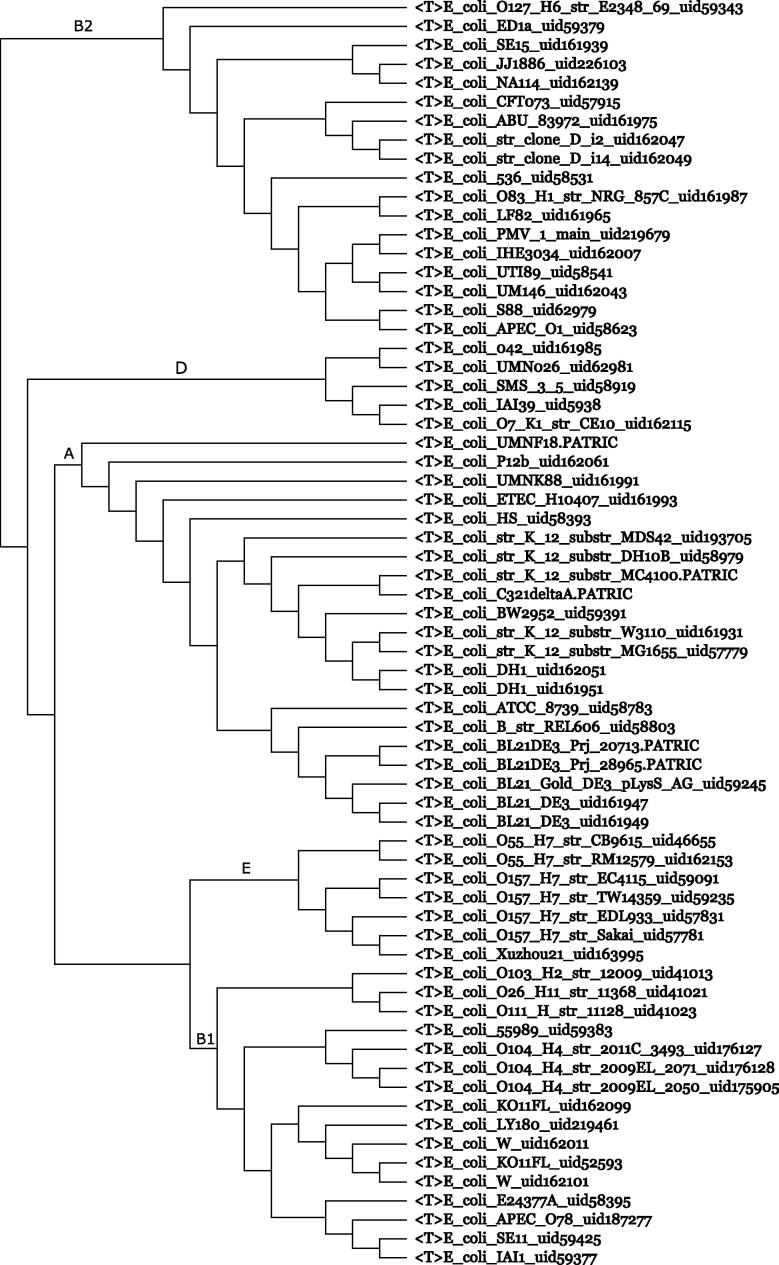
**Phylogroups of 67 *Escherichia coli* strains** The phylogroups A, B1, B2, D, and E are shown on the common branches. 〈T〉 stands for strain.

1. Serotypes of *Streptococcus pyogenes.* Figure 4 was isolated from a 3220-genome CVTree. The serotype of the 20 strains was placed in parentheses at the end of each entry, *e.g.*, M3 or M59. The branching order follows the serotype.
2. Population genetics of bacteria is an important but much less-studied subject. For example, the clonal structure of naturally occurring *Escherichia coli* communities persists despite frequent recombination events [63], [64]. Experimental methods are available for determining the phylogroups of *E. coli*, as the groups are associated with pathogenic or commensal behavior. Figure 5 shows a branch composed of 67 *E. coli* strains. The major branches in this figure correspond to the well-known phylogroups.
3. Biogeographic distribution of plants and animals lays the foundation for Darwin’s theory of evolution. However, the biogeography of bacteria has not been thoroughly examined. We refer to a recent paper [22] describing various strains of *Sulfolobus islandicus* collected from different parts of Euro-Asian and North-American continents as geovars. This work used CVTree and electronic DNA–DNA hybridization. The subtrees of *Helicobacter pylori* or *Chlamydia trachomatis* strains may be correlated with human migration patterns (figures not shown, but can be obtained from the Working Project of CVTree3).
4. As a potential application of CVTree’s high resolution power, electronic screening may be used to examine bacterial metabolic products. Screening bacterial mutant strains for pharmaceutical purposes is a costly and time-consuming process. However, when a sufficiently large amount of experimental data has been accumulated, it is easy to map new mutant genomes into a phylogenetic tree based on previous screening knowledge labeled on the branches. Thus, only promising strains are selected for further examination in laboratory screening.

## Discussion

Biology commences with taxonomy. However, the field of taxonomy as a subfield of biology, and particularly microbial taxonomy, is on the decline. Although the number of living microbial cells is estimated to be of the order of 10^30^ [1] and the number of species surely exceeds 10^6^ [65], the number of described species is only slightly above 1.1 × 10^4^ [10]. The pace of describing prokaryotic species will likely not catch up with the speed of discovery of new microorganisms. So called “minimal standards”, practiced by some editorial offices of microbiological journals [66], further hinder the valid publication of bacterial names. In the 20-th century, the International Code of Nomenclature of Bacteria [57] has played a unifying role in the field of microbial taxonomy. However, as pointed out by Barny Whitman, the supervisor of the Bergey’s Manual, “many biologists will no longer validate the names of newly described prokaryotes and the literature will once again be full of names with uncertain meaning” [66]. Fortunately, development of genome sequencing technology could help provide a solution to such situation. “With the availability of inexpensive DNA sequencing, prokaryotic species could be routinely described based upon their genome sequences” [66]. Both phylogeny and taxonomy can become by-products of genomic analysis. Reliable and easily-usable tools such as CVTree3 will play a crucial role in future development.

The whole-genome approach will not replace other methods. In contrast, we advocate for the viewpoint of polyphasic phylogeny and taxonomy. With the cost of bacterial genome sequencing dropping below that of an average phenotyping experiment, the results of phenotyping tests have become even more valuable. A tripartite comparison between whole-genome based CVTree, 16S rRNA sequences based All-Species Living Tree [67], and the Bergey’s Manual complemented by current taxonomic literature as reflected in LPSN [51] is becoming a feasible task.

## Authors’ contributions

BH initiated the CVTree approach. GZ designed and implemented the new parallelized web server. BH and GZ together tested the system and developed the biological applications of CVTree3 described in the paper. BH wrote the manuscript. Both authors read and approved the final manuscript.

## Competing interests

The authors have declared that no competing interests exist.

## Supplementary material

Supplementary material 1 CVTree3 User’s Manual.

Supplementary material 2 A representative Lineage Modification file.
