# Supplementary material for: CVTree3 Web Server for Whole-genome-based and Alignment-free Prokaryotic Phylogeny and Taxonomy
Source: Genomics Proteomics Bioinformatics. 2015 Nov 10;13(5):321–31. doi: 10.1016/j.gpb.2015.08.004 (PMC4678791; doi:10.1016/j.gpb.2015.08.004)
Supplement: Supplementary material 1 — CVTree3 User’s Manual. [file mmc1.pdf]

CVTree3 User's Manual

Guanghong ZUO and Bailin HAO

6 July 2015

Contents

1 Introduction **3**

2 Web Interface **4**

2.1 Getting Started 4
2.2 Setting Up A Project 5
2.2.1 Basic Parameters 6
2.2.2 Choosing Inbuilt Genomes 6
2.2.3 Upload Genomes 7
2.2.4 Run Project 9
2.3 Result Page 9
2.3.1 Monopoly, Collapsing, and Convergence 10
2.3.2 Summary of Taxa Monopoly with K 10
2.4 CVTree Viewer 12
2.4.1 Use of Circles and Colors 13
2.4.2 Search Query 14
2.4.3 The Taxonname {n + m } Convention 14
2.4.4 Select Node 15
2.4.5 Output Tree Figure 15
2.5 Lineage Revision and Tree Re-collapsing 16
2.5.1 Taxonomic References 16
2.5.2 Lineage Modification file 16
2.6 Example Project 19
2.7 Keep and Reload a Project 19

3 Source Code Availability **19**

4 Development History **19**

5 Acknowledgements **20**

Appendices **21**

A Inbuilt Genome Data Sets **21**
A.1 Prokaryotic Genomes 21
A.2 Eukaryote Genomes 21
A.3 Tiny Genomes 22

B Algorithm **22**
B.1 Frequency or Probability of Appearance of K-Strings 22
B.2 Subtraction of Random Background 23
B.3 Composition Vectors and Dissimilarity Matrix 23
B.4 Tree Construction 24

References **24**

1 Introduction

CVTree3 is the latest version of an effective web srver for inferring phylogeny from whole genome sequences and comparing the results with systematics at all taxonomic ranks. The alignment-free method based on K-tuple counting and background subtraction was termed a composition vector (CV) approach and the approach is abbreviated as CVTree. It was introduced in 2004 [Qi *et al.* (2004b)] with applications to prokaryote phylogeny. The first web server was published in the same year in the *Nucleic Acids Research* Web Server issue [Qi *et al.* (2004a)]. Since then it has been applied to Archaea and Bacteria [Gao *et al.* (2007); Qi *et al.* (2004b); Sun *et al.* (2010); Zuo *et al.* (2013, 2104, 2015)], viruses [Gao and Qi (2007); Gao *et al.* (2003)], chloroplasts [Yu *et al.* (2005)] and fungi [Wang *et al.* (2009); O'Connell *et al.* (2012); Choi *et al.* (2013)]. An essential update of the CVTree web server was published in the 2009 NAR Web Server issue [Xu and Hao (2009)]. The methodological aspects of the CV approach has been elucidated repeatedly in literature. In particular, the role and choice of the peptide length K was discussed in [Li *et al.* (2010)] and [Zuo *et al.* (2014)].

As the CV algorithm is CPU- and memory-demanding, the previous CVTree web servers could hardly catch up with the ever increasing amount of genomic data. Therefore, we have redesigned the data processing strategy and made the core program parallel. The new CVTree3 pipeline resides in a dedicated cluster with 64 cores. An interactive, collapsible and expandable, CVTree Viewer based on HTML5 has been added to the new web server. These new features enable biologists to study phylogeny inferred from thousands of genomes and to compare the resultis directly with taxonomy at all ranks in an almost automatic manner.

CVTree3 web server may be accessed without login requirement in many browsers, e.g., IE, FireFox, or google Chrome. However, the use of HTML5 makes IE browser lower than IE 9.0 not fully supported.

Please cite CVTree as:

1. Qi J, Wang B, Hao B L (2004) Whole proteome prokaryote phylogeny: a K-string composition approach, *J. Mol. Evol.* **58**(1): 1 – 11. [Qi *et al.* (2004b)]
2. Zuo G H, Hao B L (2015) CVTree3 web server for whole-genome-based and alignment-free prokaryotic phylogeny and taxonomy. *Genomics Proteomics & Bioinformatics*, (being submitted) [Zuo and Hao (2015)]

2 Web Interface

2.1 Getting Started

The Start Page of CVTree3 is shown in Figure 1. If a project has already been created, it can be reloaded by entering the Project Number in the textbox and clicking the “Load/Create Project’ button. To create a new project, just leave the textbox blank and click “Load/Create Project”.

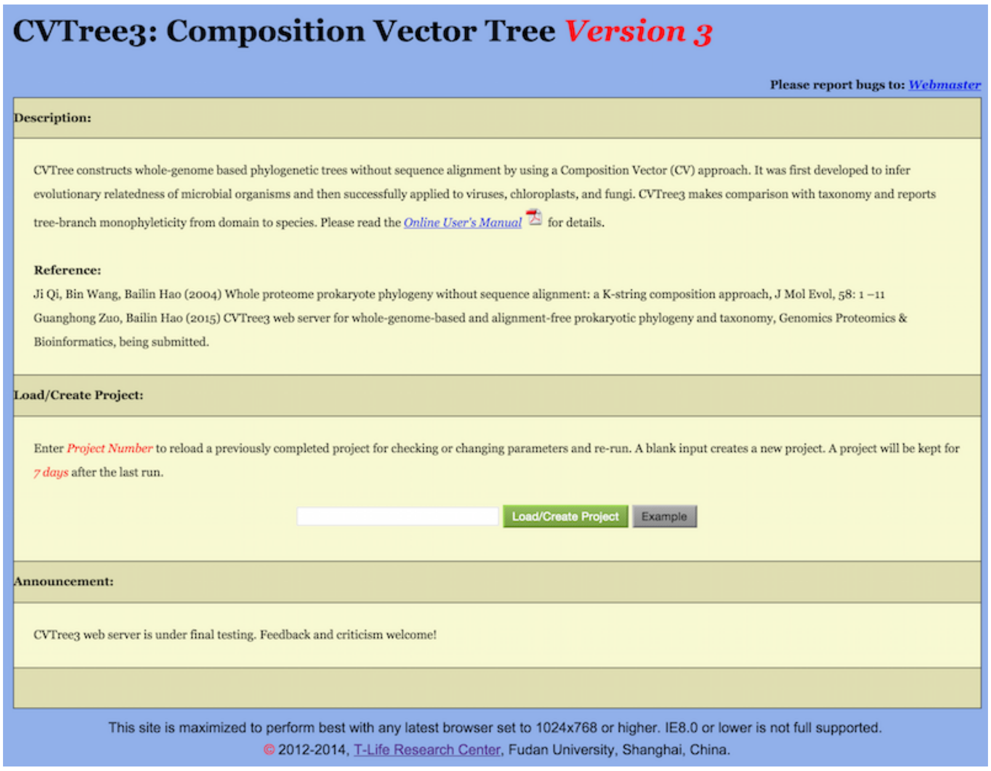

Figure 1: The Start Page of CVTree3

Each user job is assigned a unique Project Number and a work space is allocated to the project. The genomes uploaded by the user, their lineage description file with a fixed name “Lineage.txt”, as well as the Lineage Modification file, produced by the user in a working session, are stored in the work space. A project and its work space are kept for 7 days after the last run.

Next to the “Load/Create Project” button there is a buttom labeled by “Example”. By clicking at this button the system shows a preset example project. In fact, this example project is by far non-trivial. It was used in the

paper describing CVTree3 [Zuo and Hao (2015)]. First-time users are highly recommended to go through this example in order to get an overall idea on CVTree3.

2.2 Setting Up A Project

After Load/Create a project, a Setup Page in “Setting parameters” status is opened (see Figure 2). The unique Project Number shows up at the top of the page. Please keep the Project Number for subsequent reloading.

The Setup Page starts with a Project Status bar which may indicate one of three states: “Setting parameters”, “Running · · · ”, and “Project completed” (see Figure 3).

The main body of the Setup Page consists of three entitled fields, namely, “Basic Parameters”, “Select Inbuilt Genomes”, and “Upload User’s Genomes and Lineage file”.

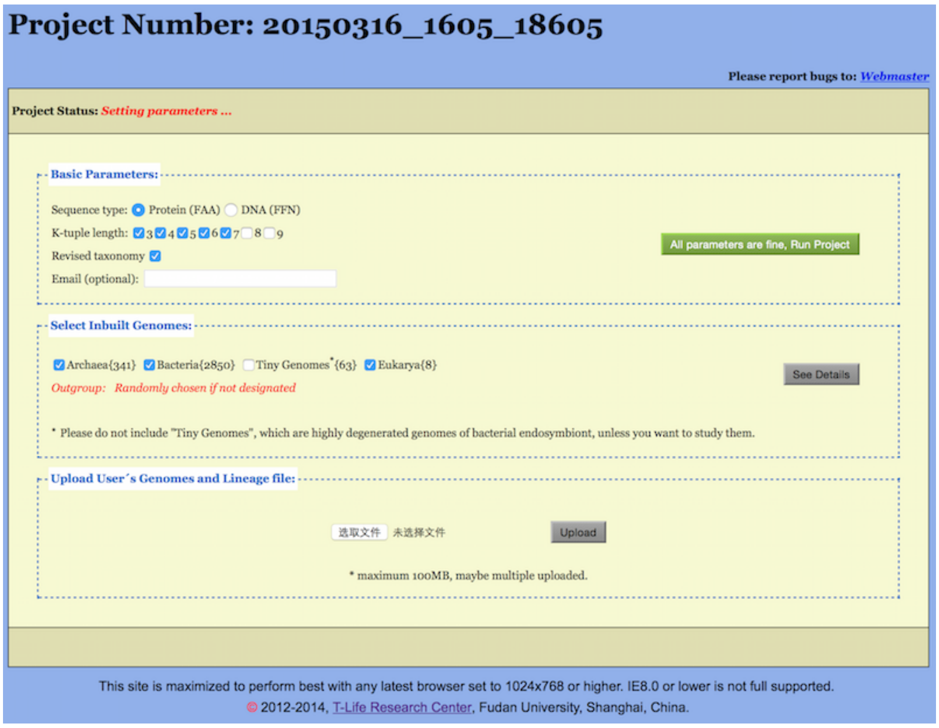

Figure 2: The Setup Page of CVTree3

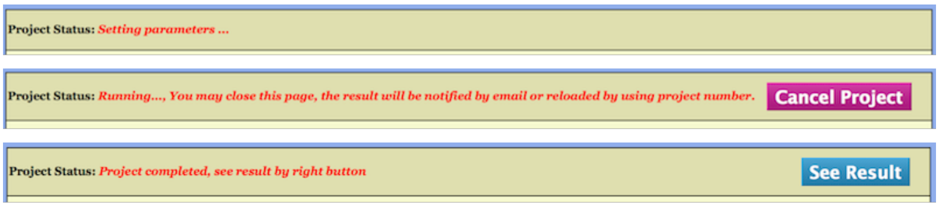

Figure 3: The status of a project

2.2.1 Basic Parameters

Basic Parameters are set by the user in the first field "Basic Parameters" of the Setup Page.

Sequence Type: Though Protein sequences are preferred, DNA sequences may be used as well.

K-tuple length: CVTree3 is capable to generate trees for all choosen K-tuple lengths in one run. The default Ks are from 3 to 7 for proteins and 6 to 18 with increment 3 for DNAs, though any single K value may be picked up. We note that when protein sequences are used the best K range is 4 and 5 for viruses, 5 and 6 for prokaryotes, 6 and 7 for fungi [Li *et al.* (2010)]. The K-values 8 and 9 are available but usually not needed.

An essentially new feature of the CVTree3 web server consists in allowing the user to compare the tree branchings with taxonomy and to see the effect of trial lineage modifications. For the Inbuilt Genomes the initial lineage information is downloaded from the NCBI taxonomy and a default taxonomy revision file is provided. A few apparently incorrect or incomplete lineages are modified in this file. If a user would not like to invoke these modifications the Revised Taxonomy box should be unchecked. Then the comparison report would solely be based on the initial NCBI taxonomy. For on about taxonomic revisions please see Section 2.5.

Optionally, a user may enter an email and be notified when the project is completed. Otherwise, the result may be reloaded at a later time using the Project Number explained above.

2.2.2 Choosing Inbuilt Genomes

The CVTree3 web server possesses a comprehensive built-in database of genomes. These genomes are subdivided into several groups as shown by the names of the selectable buttons: Archaea(341), Bacteria(2850), Tiny Genomes(63), and Eukarya(8). Numerals in parentheses indicating the number of genomes in each group as of 1 January 2014. For more about these

genome groups, please see the Appendix A.

By clicking the checkbox in front of each group, one can select or unselect a whole group. The outgroup will be chosen by the web server “at random”.

If one wants to select genomes one by one and set an outgroup by oneself, please get into the Select Inbuilt Genomes Page, shown in Figure 4, by clicking on the “See Details” button.

This page consists of a long list of all builtin genomes. Entries in this table is sortable by clicking on the head of the table. For example, by clicking on “Genome name” all the genomes would appear in alphabetic order of their names; by clicking on “Proteome” the genomes will be ordered by the total number of amino acids in fraction of M (10^6) (this is so even when Sequence type on the previous page was chosen to be DNA (FFN)), just giving an idea about the genome size.

The first column ”Out-group” is a toggle switch. Only one entry may be selected while all others be unselected.

The last column of the table possesses a pull-down list of taxonomic ranks with number of taxa in each rank: from Domian{3} to Species{3191} for the time of writing these lines. A user can pick up an item from the list to facilitate the selection of genomes.

After completing the selection one returns to the Project Set page (Figure 2) by clicking on “Done & Back to Project page”.

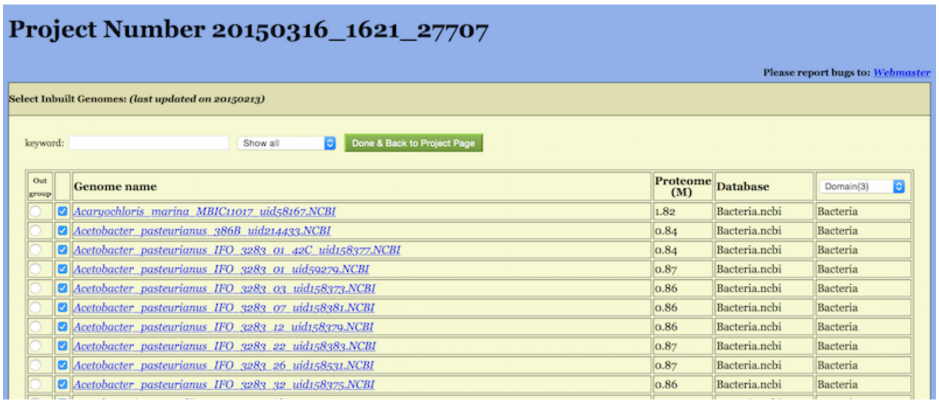

Figure 4: Select Inbuilt Genomes Page

2.2.3 Upload Genomes

In the functional field “Upload Genomes” a user can upload one’s own multifasta files with the extension .faa for protein sequences or .ffn for the DNA

sequences (see Figure 5). A list of all uploaded genomes appears in the field, but only checked ones are used when the project is submitted for running. Note that all genomes are selected by default when uploaded. All user genomes, checked or unchecked, together with the configured project will be kept for 7 days after the last run.

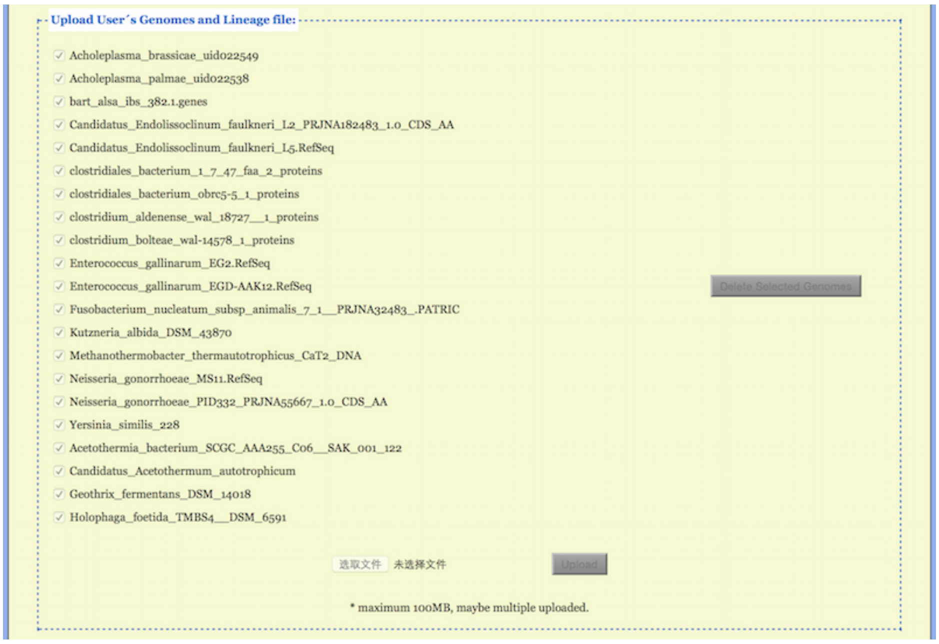

Figure 5: Upload Genomes

User may prepare and upload a lineage information file with a fixed name “Lineage.txt” for the uploaded genomes. Information for each genome takes a line as

<D>Bacteria<K>Bacteria<P>Proteobacteria<C>Gammaproteobacteria
<O>Enterobacteriales<F>Enterobacteriaceae<G>Yersinia<S>Yersinia_similis

Here <D>, <K>, <P>, <C>, <O>, <F>, <G>, <S> and <T> stand for Domain, Kingdom, Phylum, Class, Order, Family, Genus, Species and sTrain, respectively. Incomplete lineage information may appears as, e.g., <O>Unclassified<F>Unclassified. Note that “Unclassified” is a standard classifier used in the lineage information. If the Lineage.txt file or some lines in it are lacking, the CVTree web server complements them with Unclassified ranks. User may add or change lineage information during a session using the Lineage Modification function to be described later.

Actually, no taxonomic information is required when uploading user-supplied genomes, because one would like to use CVTree3 to predict taxonomic position of newly sequenced genomes. In the CVTree Viewer if the cursor is put on such a genome name the lineage information would look like

<D>Unclassified<K>Unclassified<P>Unclassified$\cdots$<G>Unclassified$\cdots$

Such genomes are not counted in checking monopoly and in generating convergence statistics. However, their number appears as an addend in a collapsed leaf in the tree viewer, see subsection 2.4.3.

2.2.4 Run Project

When all paramenters are set the project is submitted for processing by clicking the button "All parameters are fine, Run Project". After submission the "Setting parameters" status will be locked and the project status changes to that shown in the middle of Figure 3, namely, "Running $\cdots$". The project will be done in a few minutes if only the inbuilt genomes are used. If many new genomes are uploaded, the waiting time might be much longer, depending on the size and number of genomes. One can safely close the page and the completion of job be notified later by email if an email has been entered when setting parameters. Otherwise, one may revisit the web server and reload the project by entering the Project Number.

If necessary one can cancel the project and reset parameters by clicking the button "Cancel Project".

2.3 Result Page

When the project is completed, the Project Status bar changes to that shown in the bottom of Figure 3. By clicking on the "See Result" button one is led to the Result Page.

The Result Page almost entirely consists of a long table summarizing taxa convergence except for two buttons in the upper-right corner: "See Tree" and "Download Result".

"See Tree" is the portal to the interactive tree display to be described in Section 2.4.

"Download Result" is where the user can download the results to the local computer for further analysis and archiving. It may be used any time while on-line or after reloading a project.

2.3.1 Monopoly, Collapsing, and Convergence

A prominent feature of the CVTree approach consists in that the resulted trees are justified by direct comparison with taxonomy rather than by statistical resampling tests such as bootstrap or jackknife. Statistical resampling tests tell at most the stability and self-consistency of the tree with respect to small variations of the input data, by far not the objective correctness of the trees. We note, nevertheless, the CVTree results have also successfully passed various statistical resampling tests [Zuo *et al.* (2010)].

A central notion in comparing a tree with taxonomy is monopoly. The notion of monopoly applies to phylogeny as well as to taxonomy, see, e.g., discussion by James Farris [Farris (1974, 1990)]. However, we use it in a pragmatic way by restricting to the classification of genomes in the input dataset and to the collection of genomes in various tree branches.

If all genomes in a certain tree branch come from one and the same taxon and no genomes from other taxa having mixed in, the branch is said to be monophyletic at this taxonomic rank. For example, this happens to Cyanobacteria as all the 77 genomes designated to this phylum in the input dataset appear entirely and exclusively in one and the same branch. Now the branch may be fully collapsed into one leave labeled by Cyanobacteria{77}. In this way, the total number of leaves seen in a tree may be greatly reduced.

From a taxonomic point of view a taxon is monophyletic only when all species listed in it are descendants of one and the same ancester. As this is a hardly provable fact, monphyleticity has to be deduced from some phylogenetis study. For example, according to vol. 3 of The Bergey's Manual, 77 species out from 167 listed in the genus Clostridium form a cluster in a 16S rRNA gene tree. These are considered members of Costridium *senso stricto*, whereas the remaining 90 species are distributed in 10 different clusters. Naturally, one cannot expect a monophyletic branch of Clostridium genomes for the time being.

When a branch is collapsed monophyletically to a leave made of genomes from one and the same taxon, it is said to be convergent at this taxonomic rank. In other words, only when collapsing leads to a monophyletic leave, the taxon is considered convergent at the corresponding K. Usually this happens at one or more K-values. Convergence at most or all K-values adds confidence to the result, although the branching topology may be slightly different.

2.3.2 Summary of Taxa Monopoly with K

Convergence of taxa at various K-values provides an additional angle to look at the phylogeny. That is why CVTree3 calculates trees at several Ks in one

run and produces a summary report.

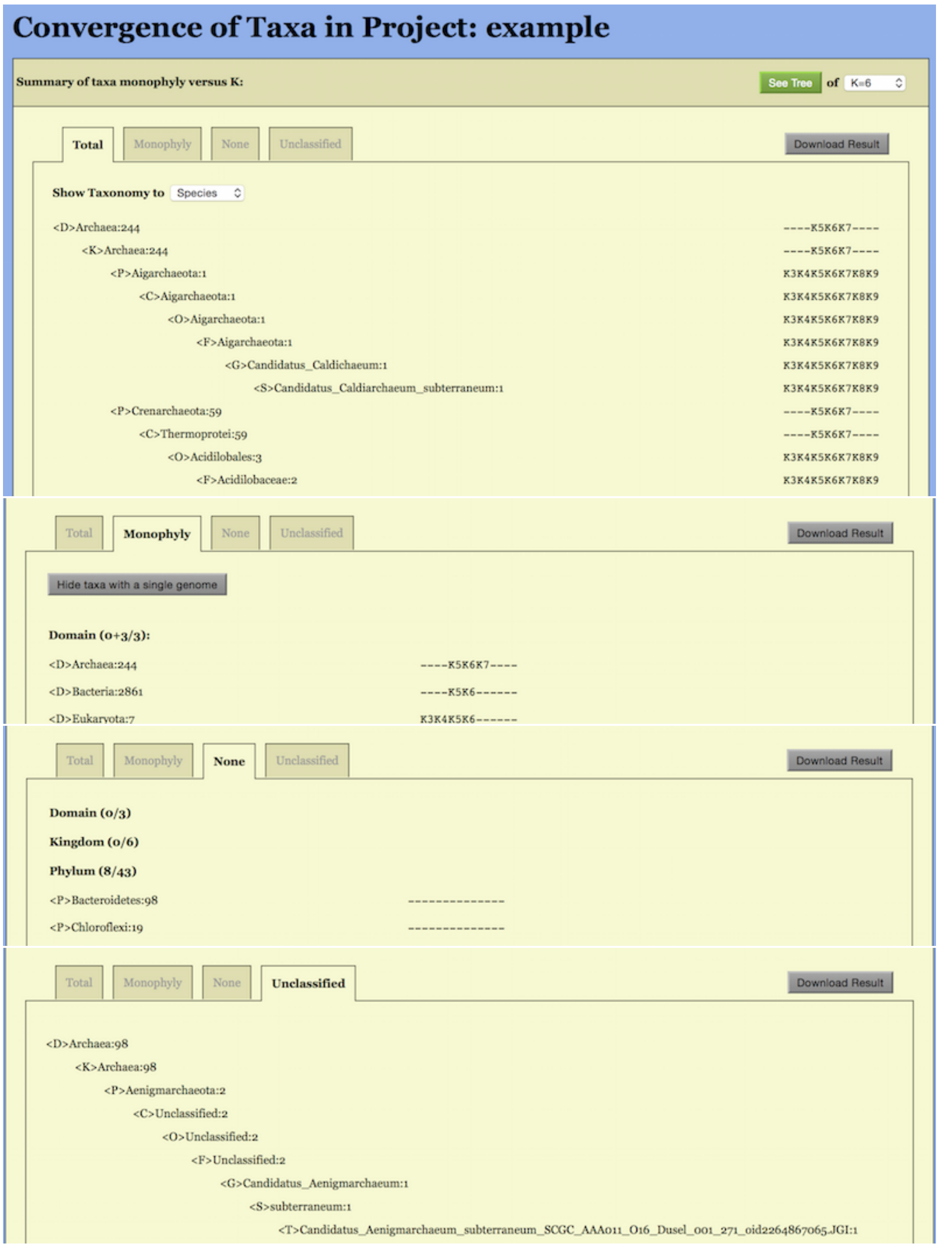

Figure 6: The Taxa Convergence Summary

The taxa convergence summary may be displayed in four ways: “Total”, “Monophyly”, “None” and “Unclassified”.

The “Total” way generates an alphabetic list of all taxa in taxonomic

order. Abbreviations are used for the ranks: <P> Phylum, <C> Class, <O> Order, <F> Family, <G> Genus, and <S> Species. The same set of abbreviations with an additional <T> for sTrains are used in the CVTree Viewer.

A typical page is shown in the top part of Figure 6. A taxon name is followed by the number of genomes belonging to the taxon. One can choose to show the list from Domain down to a certain taxonomic rank, e.g., Family, by setting "Show Taxonomy to Family". The monophyly status of the taxon is given at the right. For example, the archaeal class Halobacteria is represented by 27 genomes and it is monophyletic at all K's except for $K = 4$, then the corresponding line in the list reads:

<C> Halobacteria: 27 K3- -K5K6K7- - - -

The option "Monophyly" lists taxa which are monophyletic at least for one K, as shown in the middle of Figure 6. If one would like to see a list of taxa which are not monophyletic for all K, the option "None" serves for this purpose, see the lowest part of Figure 6.

When "Monophyly" is chosen, each taxonomic rank as a subtitle carries a statistic. For example, a line

Class (20 + 34 /65)

tells that the total number of classes is 65 in the input dataset (after taking into account taxonomic revision, if any); among these 65 classes 20 are represented by only one genome hence are trivially monophyletic; the other 34 are represented by two or more genomes and are monophyletic at least for one K.

In the "None" option, the corresponding line reads

Class (11/65)

indicating that there are $65-20-34 = 11$ classes, which are not monophyletic for whatsoever K. In a sense, these non-monophyletic taxa are worth further studying as they may hint on possible taxonomic revisions.

If "Unclassified" option is chosen, a list of taxa with at least one Unclassified rank would be shown. This helps to look for taxa with incomplete lineage information.

2.4 CVTree Viewer

It is extremely difficult, if not impossible, to comprehend a tree made of thousands of leaves. To this end an interactive, collapsible and expandable, display has been developed for the CVTree3 web server. As skillful manipulation of the display is the key point to make the most of CVTree3. Therefore, we explain the interactive display in more details.

By clicking the button "See Tree" in the upper-right corner of the Result

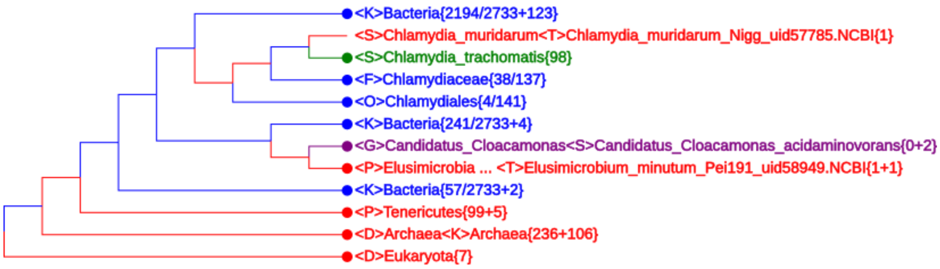

Figure 7: A typical page of CVTree Viewer

Page (see Figure 6), a CVTree Viewer page with default $K = 6$ opens up. A typical tree, plotted by using HTML5, is shown in Figure 7.

First of all, in Figure 7 all 342 Archaea, 2870 Bacteria and 7 Eukarya genomes are accomodated within a single screen. The {n + m} convention in number of genomes will be explained later.

This picture was obtained after searching for the species name *Chlamydia_trachomatis* and only its neighborhood has been expanded somehow, while all other branches were maximally collapsed except for one line exposed in purple. In total, four colors are used in this figure. Their meaning will be explained in the next subsection.

2.4.1 Use of Circles and Colors

If a node is denoted by a blank circle (○), it is collapsible. One may click on the circle to have all the lower branchings shrinked; the collapsed branch is labeled by the highest-rank common taxon name. At the lowest level, a rightmost node may be marked by a solid circle (●) preceding a taxon name; it tells that there are more than one genomes in that branch and it may be expanded by clicking on the solid circle. In contrast, a short line (—) in place of a would-be circle means that there is only one genome and it cannot be further expanded.

Taxon names may appear in one of four colors: red, blue, green and purple. A red name indicates that the branch is monophyletic and collapsed. This includes the trivial case when a taxon is represented by a single genome.

A collapsed but not convergent branch such as Chlamydiaceae{38/137} is shown in blue. In this particular case, the other 137 - 38 = 99 genomes are located in the two lines above. Taken together, they would yield a monophyletic family Chlamydiaceae{137} shown in red. A taxon name in green matches the word a user types in place "Search Query", i.e., Chlamydia_trachomatis in this picture.

Purple color is used to show a taxon with incomplete lineage information. In Figure 7 there is only one line in purple:

<S>Candidatus_Cloacamonas_acidoaminovorans{0+2}

corresponding to two genomes belonging to a newly proposed phylum without legal standing in nomenclature.

2.4.2 Search Query

For the best K value 5 or 6 the CVtree Viewer first opens in a maximally collapped state with 3 leaves: •<D>Bacteria<K>Bacteria{2861 + 9}, •<D>Archaea <K>Archaea{244 + 98}, and •<D>Eukaryota{7}, representing the three main domains of life and witnessing the correctness of Carl Woese. The meaning of {2861 + 9} and {244 + 98} will be explained in the next subsection.

The quickest way to get to the point of interest in a tree is typing a taxon name in place of the "Search Query". For example, one may type Chlamydia and select an item from the pull-down list, e.g., Chlamydia_trachomatis, the bacterium first isolated and cultured as a virus by the outstanding Chinese microbiologist Feifan Tang [Tang *et al.* (1957)]. In the CVTree display the line <S>Chlamydia_trachomatis{98} is highlighted in green while all other branches being maximally collapsed, as shown in Figure 7.

One can pick up a branch and let it fills up the whole display window. For example, to single out the species <S>Chlamydia_trachomatis{98} one holds the shift key and clicks on the solid circle in front of the species name. The leaf •<S>Chlamydia_trachomatis{98} will move to the leftmost position in the display and further clicking on the solid circle expands it to the whole window.

2.4.3 The Taxonname {n + m } Convention

A taxon name such as Bacteria{2861 + 9} indicates that 9 of the 2870 bacterial genomes did not come with complete lineage information. Only taxa with complete lineage information are counted in the convergence report as an augend {n +, while genomes without complete lineage information are indicated in the tree display as an addend +m}. This convention is useful for studying taxonomic assignment of newly sequenced genomes without proper lineage information. However, please note that lineage information for a given taxon may be complete but incorrect, thus requiring further modification.

2.4.4 Select Node

The aforementioned operation of picking up a branch may be performed in another way, namely, by using the “Select Node” option in the headline of the CVTree Viewer. By selecting a taxon name in the pull-down list, e.g., <P>Aquificae{14}, the phylum *Aquificae* represented by 14 genomes for the time being is displayed in the whole window. By selecting an higher taxon, e.g., <D>Bacteria{2861} the display restores to that for the whole domain Bacteria.

2.4.5 Output Tree Figure

When a tree view has been adjusted by appropriately collapsing and expanding, a print quality figure can be obtained by clicking on the “output” button in the upper-right corner of the CVTree Viewer page (Figure 7). It opens an output preview page (Figure 8).

One may select a format to save a figure. The default format is SVG (Scalable Vector Graphics), as the underlying plot is done in SVG. Before saving, a figure may be monochromatized. One may choose PDF, eps, and png formats as well. If a user wishes to modify the output figure, say, by adding texts or changing color, the SVG format is recommended, especially, when convenient SVG tools such as Inkscape is available.

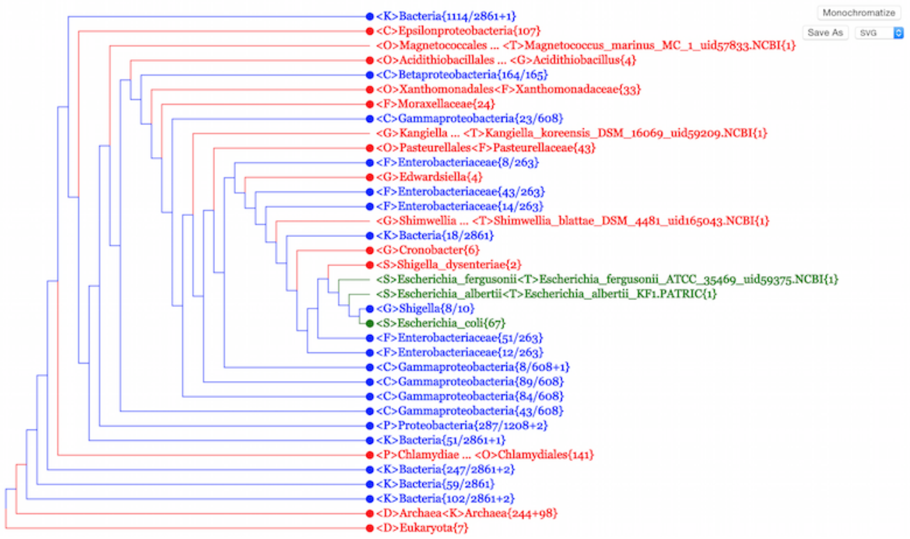

Figure 8: The Output Preview Page of CVTree3

Please do not forget to quit the preview page in order to continue working

with the CVTree Viewer.

2.5 Lineage Revision and Tree Re-collapsing

Now we arrive at another prominent feature of CVTree approach, namely, justification of the resulted trees by direct comparison with taxonomy, instead of performing statistical re-sampling tests such as bootstrap or jackknife. For prokaryotes direct comparison with taxonomy has become feasible only quite recently. On one hand, the completion of the second edition of *The Bergey's Manual of Systematic Bacteriology* [Bergey's Manual Trust (2012)], which has been considered by many microbiologists as the best approximation to an official classification [Konstntinidis and Tiedje (2005)], provides a state-of-the-art framework for taxonomy together with current literature such as as IJSEM *International Journal of Systematic and Evolutionary Microbiology*. On the other hand, the development of the CVTree approach has provided prokaryotic phylogeny a convenient and comprehensible platform [Li *et al.* (2010); Hao (2011)].

2.5.1 Taxonomic References

Speaking about taxonomy one must admit that there is no generally accepted standard for prokaryotic taxonomy. The temptation to become a standard makes the Bergey's systematics a more conservative source. For example, there were deadlines and other restrictions for inclusion in The Manual. Many newly sequenced genomes do not have neither a standing in bacterial nomenclature nor a validly published name. These organisms are not reflected in Bergey's Manual or in current literature. In contrast, the NCBI taxonomy, though disclaimed to be a taxonomic reference, is, in fact, more dynamic and up-to-date. At least, for any sequence deposited into GenBank there is a piece of lineage information in NCBI taxonomy, no matter how incomplete it might be. Therefore, CVTree3 takes initial lineage information from NCBI.

2.5.2 Lineage Modification file

In order to see the initial lineage information one should uncheck the Revised taxonomy box in the Setup Page (Figure 2) during parameter setting. Then in the “Unclassified” part of the Convergence of Taxa table in the Result Page one might see many lines like

· · · <G>Unclassified<S>Unclassified<T>Uncultured_archaeon_A07HR67· · ·
· · · <G>Unclassified<S>Unclassified<T>halophilic_archaeon_True_ADL· · ·
· · · <G>Unclassified<S>Unclassified<T>Uncultured_archaeon_A07HN63· · ·
. . . .

Clearly, these are organisms without a proper taxonomic assignment and lacking validly published names. In fact, by moving the cursor in the tree display to this name, a pull-down window with lineage information appears for a few seconds. One sees lineages with "Unclassified" ranks as shown above.

However, CVTree clearly shows all these organisms belong to the order *Halobacteria*, which contains a single family *Halobacteriaceae*. One would like to see the effect of making appropriate lineage revisions. The option "Lineage Modificaion" in the CVTree Viewer provides this function. By clicking on this option an empty "Lineage Modification" window opens up: it looks like Figure 9 but without any text.

In order to modify a lineage one simply writes a line with the old lineage followed by the new modified lineage separated by a space, i.e.,

old_lineage new_lineage

The Example project comes with an example Lineage Modification file. We refer to this file for details of writing lineage modification information. Please note that in this file anything after a "#" sign means a comment. A few brief references are given in these comments. In particular, some linage modifications are associated with archaeal genomes from the Microbial Dark Matter project [Rinke *et al.* (2013)].

When a Lineage Modification file is ready, one clicks on the "Submit" button in the bottom line. The system shows "Recollapsing is running. Please wait.“ It takes a minute or two. Then it says "Recollapse successfully". Both the taxa convergence table and the CVTree Viewer have been renewed.

As there are many explicit taxonomic problems in the initial NCBI information, the example Lineage Modification file also serves as a default file. If the Revised taxonomy box is checked in the beginning the default file will be used for comparison with taxonomy; otherwise, the initial NCBI information is used. Alternatively, one may use the buttons "Clear Text", "Reset", "Default", "Save", and "Submit", to manage the lineage modification process. For example, "Clear Text" makes the Lineage Modification window empty and "Default" restores it to the default file. An user-generated Lineage Modificaion file, saved to the local computer, may be used in subsequent new projects.

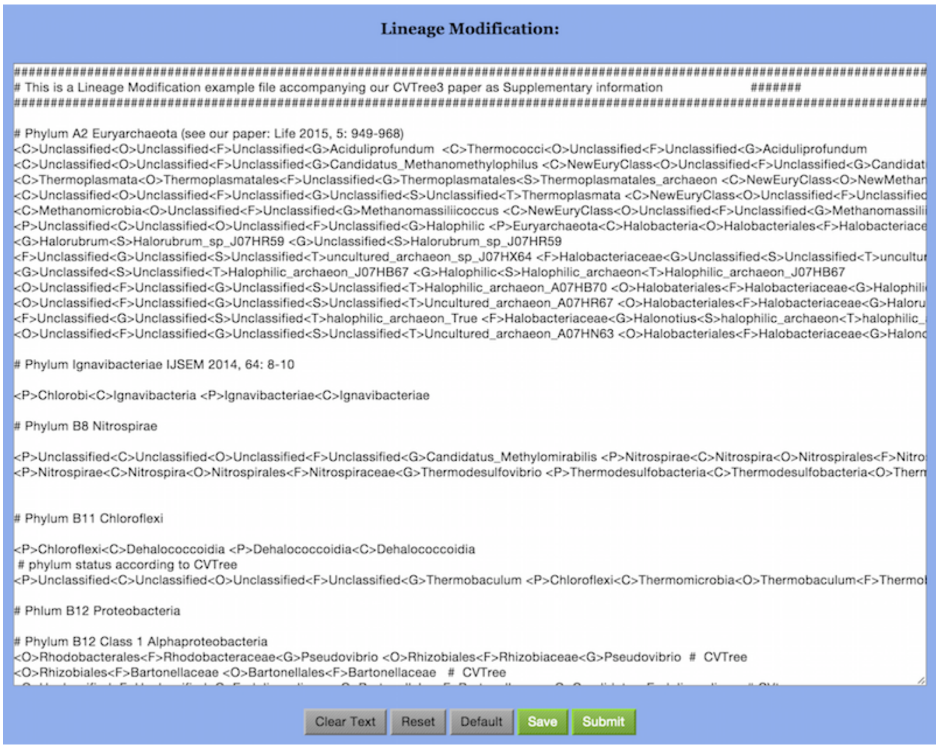

Figure 9: An example Lineage Modification file

Without going into details, we indicate that using the initial NCBI information one would get at the top level
Archeae{231 + 111} and Bacteria{2725 + 145}.

With the example Lineage Modification file used one would get

Archeae{235 + 107} and Bacteria{2733 + 137}.

A detailed comparison of the differences is very instructive for understanding the lineage modification mechanism. In particular, the change from 111 to 107 in the Archaea part led to two monophyletic genera *Halorubmrum*{*4*} and *Halonotius*{*4*}. It demonstrates the potential of using CVTree to bring prokaryotic taxonomy to a better shape.

However, we emphasize that actual taxonomic revisions must comply with the International Code of Nomenclature of Bacteria [Lapage *et al.* (1992)] and follow the established practice in the microbiological community. The Lineage Modificarion function provided by CVTree3 is solely for trial purpose.

2.6 Example Project

In order to help the users to acquaint quickly with the new features of the CVTree3 web server a pre-set Example project is provided. One simply invokes the example project by clicking on the "Exmaple" button in the Start Page (see Figure 1). One can watch the result of this Example project by clicking the buttons as described before, but cannot get the project modified and re-running.

2.7 Keep and Reload a Project

The unique Project Number assigned at setting up a new project is used for reloading the project at a later time. After reloading one may adjust the parameters and rerun the job. A project is kept for 7 days after the last run. What kept includes

1. The parameter setting.
2. The user uploaded genomes.
3. The Taxonomic Revision file, usually produced by modifying the default file.

3 Source Code Availability

For academic users who are interested in the inner workings of the CVTree algorithm we can provide a stand-alone CVTree program. It is capable to calculate CVs and dissimilarity between CV pairs in comand window mode. It does not contain web interface, automatic updating machinery, and parallelization. To get a free copy of the source code with a manual please write to Dr. Guanghong Zuo at ghong.zuo at gmail.com with your full name and affiliation indicated.

4 Development History

The CV approach was first announced in 2002 at C. N. Yang's 80th Birthday Conference [Hao *et al.* (2003)] and applied to coronovivuses [Gao *et al.* (2003)] and prokaryotes [Qi *et al.* (2004b)]. Stand-alone CVTree programs were written from scratch by Ji Qi, Lei Gao, Jiandong Sun, Zhao Xu, and Guanghong Zuo, independently at different times. The first CVTree web server was built by Ji Qi and Hong Luo in 2004 [Qi *et al.* (2004a)]. An essentially improved update was constructed by Zhao Xu in 2009 [Xu and Hao

(2009)]. The present parallelized CVTree3 web server has been implemented by Guanghong Zuo since 2012 and tested by many colleagues.

5 Acknowledgements

The CVTree project has been supported by National Basic Research Project of China (973 Programs No. 2007CB814800 and No. 2013CB834100), and by the State Key Laboratory of Applied Surface Physics and Department of Physics, Fudan University.

Appendices

A Inbuilt Genome Data Sets

A.1 Prokaryotic Genomes

As the NCBI [NCBI Resource Coordinators (2013)] FTP site basically stopped releasing prokaryotic genomes since the beginning of 2014, the CVTree3 web server has to change the monthly updating policy. Genomes are collected from various resources including the following

1. GenBank, Genomes, RefSeq adabases at NCBI:
   `ftp://ftp.ncbi.nih.gov/`
2. Genome pages at EBI:
   `http://www.ebi.ac.uk/genomes/index.html/`
3. Integrated Microbial Genomes at DOE Joint Genome Institute:
   `http://img.jgi.doe.gov/`
4. The Broad Institute:
   `olive.broadinstitute.org/`
5. Pathosystems Resource Integrated Center (PATRIC):
   `http://patricbrc.or/`

Please note that many genomes from the Microbial Dark Matter project [Rinke *et al.* (2013)] have been included in the CVTree3 built-in dataset. Most of these organisms do not have proper taxonomic assignment and validly published names. They make most of the 111 genomes in Archaea$\{231 + 111\}$. Their inclusion aims to facilitate further study.

As of 31 March 2015 there were in total 3262 genomes in the CVTree3 built-in dataset, including 2913 Bacteria, 341 Archaea, and 8 Eukaryotes.

A.2 Eukaryote Genomes

Eight eukaryotic genomes, 4 fungal and 4 non-fungal, are provided for serving as outgroup in tree construction. The non-fungal genomes are *Caenorhabditis elegans*, *Arabidopsis thaliana*, *Plasmodium falciparum* and *Drosophila melanogaster*.

Historically, there were more fungal genomes given in the built-in dataset, because CVTree was applied to construct a fungal phylogeny based on 82 genomes [Wang *et al.* (2009)]. These have been removed from CVTree3.

A.3 Tiny Genomes

There are a few highly degenerated genomes of bacterial endosymbiont bacteria in the in-built database. Their proteomes are very small (< 10000 amino acids), hence the adjective "Tiny". Due to lacking of many genes the position of these species in the phylogeny often turns out to be questionable, e.g., they tend to the root and occasionally violate the trifurcation of the three main domains of life. This is why we suggest not to include the "Tiny Genomes" in a study of mostly "free-living" organisms.

On the other hand, if one is interested in these highly degenerated genomes, then it should be reminded that the cut-off at 10^4 amino acids is artificial and many slightly larger genomes, i.e., those from some insect symbionts in the family *Enterobacteriaceae* must be taken into account as well.

B Algorithm

Since the CVTree method has been described many times in the literature [Qi *et al.* (2004b); Li *et al.* (2010); Hao (2011)], here we only present a brief overview.

B.1 Frequency or Probability of Appearance of K-Strings

The alignment-free way of genome comparison is realized by extending single nucleotide or single amino acid counting to that of longer K-strings. Among early work along this line we mention the use of dinulceotide relative abundance as a genomic signature [Karlin and Burge (1995)]. Given a DNA or amino acid sequence of length L, we count the number of appearance of (overlapping) strings of a fixed length K in the sequence. The counting may be performed for a complete genome or for a collection of translated amino acid sequences. There are in total N possible types of such strings: $N = 4^K$ for DNA and $N = 20^K$ for amino acid sequences.

For concreteness consider the case of one protein sequence of length L. Denote the frequency of appearance of the K-String $a_1a_2\cdots a_K$ by $f(a_1a_2\cdots a_K)$, where each a_i is one of the 20 amino acid single-letter symbols. This frequency divided by the total number $(L-K+1)$ of K-Strings in the given protein sequence may be taken as the probability $p(a_1a_2\cdots a_K)$ of appearance of the string $a_1a_2\cdots a_K$ in the protein:

$$p(a_1a_2\cdots a_K) = \frac{f(a_1a_2\cdots a_K)}{(L-K+1)} \qquad (1)$$

The collection of such frequencies or probabilities reflects both the result of random mutations and selective evolution in terms of K-strings as building blocks.

B.2 Subtraction of Random Background

Mutations happen in a more or less random manner at the molecular level, while selections shape the direction of evolution. Neutral mutations lead to some randomness in the K-string composition. In order to highlight the selective diversification of sequence composition one must subtract a random background from the simple counting results. This is done as follows.

Suppose we have done direct counting for all strings of length $(K-1)$ and $(K-2)$. The probability of appearance of K-strings is predicted by using a Markov model:

$$p^0(a_1a_2\cdots a_K) = \frac{p(a_1a_2\cdots a_{K-1})p(a_2a_3\cdots a_K)}{p(a_2a_3\cdots a_{K-1})} \tag{2}$$

The superscript 0 on p^0 indicates the fact that it is a predicted quantity. We note that the denominator comes from the frequency of $(K-2)$-strings. This kind of Markov model prediction has been used in biological sequence analysis since long [Brendel *et al.* (1986)]. It can be justified by virtue of a maximal entropy principle with appropriate constraints [Hu and Wang (2001)].

B.3 Composition Vectors and Dissimilarity Matrix

It is the difference between the actual counting result p and the predicted value p^0 that really reflects the shaping role of selective evolution. Therefore, we collect

$$a_i(a_1a_2\cdots a_K) = \begin{cases} \frac{p(a_1a_2\cdots a_K)-p^0(a_1a_2\cdots a_K)}{p^0(a_1a_2\cdots a_K)} & \text{when } p^0 \neq 0 \\ 0 & \text{when } p^0 = 0 \end{cases} \tag{3}$$

for all possible strings $a_1a_2\cdots a_K$ as components to form a composition vector for a species. To further simplify the notations, we write a_i for the i-th component corresponding to the string type i, where i runs from 1 to $N = 20^K$. Putting these components in a fixed order, we obtain a composition vector for the species A:

$$A = (a_1, a_2, \cdots, a_N)$$

Likewise, for the species B we have a composition vector

$$B = (b_1, b_2, \cdots, b_N)$$

In principle there are different ways to construct the composition vectors. First, one may use the whole genome sequence. Second, one may just collect the coding sequences in the genome. Third, one makes use of the translated amino acid sequences from the coding segments of DNA. As mutation rates are higher and more variable in non-coding segments and protein sequences change at a more or less constant rate, one expects that the third choice is the best and the second is better than the first. We tried all three choices and the requirement of consistency served as a criterion. By consistency we mean the topology of the trees constructed with growing K should converge. This is best realized with phylogenetic relations obtained from protein sequences. Therefore, in what follows we concentrate on results based on amino acid sequences.

The correlation $C(A,B)$ between any two species A and B is calculated as the cosine function of the angle between the two representative vectors in the N-dimensional space of composition vectors:

$$C(A,B) = \frac{\sum_{i=1}^{N} a_i \times b_i}{(\sum_{i=1}^{N} a_i^2 \times \sum_{i=1}^{N} b_i^2)^{\frac{1}{2}}} \tag{4}$$

The distance $D(A,B)$ between the two species is defined as

$$D(A,B) = \frac{1 - C(A,B)}{2} \tag{5}$$

Since $C(A,B)$ may vary between -1 and 1, the distance is normalized to the interval $(0,1)$. The collection of distances for all species pairs comprises a dissimilarity matrix. We prefer dissimilarity to distance, because the $D(A,B)$ defined above does not guarantee the fullfilment of all triangle inequalities [Li *et al.* (2010)].

B.4 Tree Construction

Once a distance matrix has been calculated it is straightforward to construct phylogenetic trees by using the neighbor-joining (NJ) method [Saitou and Nei (1987)].

References

Bergey's Manual Trust (2001-2012). *The Bergey's Manual of Systematic Bacteriology*. Springer-Verlag, New York, Heidelberg, second edition.

Brendel, V., Beckmann, J. S., and Trifonov, E. N. (1986). Linguistics of nucleotide sequences: morphology and comparison of vocabularies. *Journal of Biomolecular Structure & Dynamics*, **4**(1), 11–21. PMID: 3078230.

Choi, J., Kim, K. T., Jeon, J., and Lee, Y. H. (2013). Fungal plant cell wall-degrading enzyme database: a platform for comparative and evolutionary genomics in fungi and Oomycetes. *BMC Genomics*, **14(Suppl. 5)**, 57.

Farris, J. S. (1974). Formal definitions of paraphyly and polyphyly. *Systematic Zoology*, **23(4)**, 548–554.

Farris, J. S. (1990). Haeckel, History, and Hull. *Systematic Zoology*, **39(1)**, 81–88.

Gao, L. and Qi, J. (2007). Whole genome molecular phylogeny of large dsDNA viruses using composition vector method. *BMC Evolutionary Biology*, **7**, 41.

Gao, L., Qi, J., Wei, H. B., Sun, Y. G., and Hao, B. L. (2003). Molecular phylogeny of coronaviruses including human SARS-CoV. *Chinese Science Bulletin*, **48**(12), 1170–1174.

Gao, L., Qi, J., Sun, J. D., and Hao, B. L. (2007). Prokaryote phylogeny meets taxonomy: An exhaustive comparison of composition vector trees with systematic bacteriology. *Science in China Series C: Life Sciences*, **49**(5), 587–599.

Hao, B. L. (2011). CVTrees support the Bergey's systematics and provide high resolution at species level and below. *Bulletin of BISMiS*, **2,Part 2**, 189–196.

Hao, B. L., Qi, J., and Wang, B. (2003). Prokaryote phylogeny based on complete genomes without sequence alignment. *Modern Physics Letters B*, **17**, 91–94.

Hu, R. and Wang, B. (2001). Statistically significant strings are related to regulatory elements in the promoter regions of Saccharomyces cerevisiae. *Physica A: Statistical Mechanics and its Applications*, **290**(3-4), 464–474.

Karlin, S. and Burge, C. (1995). Dinucleotide relative abundance extremes: a genomic signature. *Trends in Genetics: TIG*, **11**(7), 283–90. PMID: 7482779.

Konstntinidis, K. T. and Tiedje, J. V. (2005). Towards a genome-based taxonomy for prokaryotes. *Journal of Bacteriology*, **187**, 6258–6264.

Lapage, S. P., Sneath, P. H. A., and Lessal, E. F. (1992). *International Code of Nomenclature of Bacteria: Bacteriological Code 1990*. ASM Press, Washington, DC.

Li, Q., Xu, Z., and Hao, B. L. (2010). Composition vector approach to whole-genome-based prokaryote phylogeny: success and foundations. *Journal of Biotechnology*, **149**, 115–119.

NCBI Resource Coordinators (2013). Database resources of the national center for biotechnology information. *Nucleic Acids Research*, **41**, D8–D20.

O'Connell, R. J., Thon, M. R., Hacquard, S., and Amyotte, S. G. (2012). Lifestyle transition in plant pathogenic collectotrichum fungi deciphered by genome and transcriptome analyses. *Nature Genetics*, **44**, 1060–1065.

Qi, J., Luo, H., and Hao, B. (2004a). CVTree: a phylogenetic tree reconstruction tool based on whole genomes. *Nucleic acids research*, **32**(Web Server issue), W45–7. PMID: 15215347.

Qi, J., Wang, B., and Hao, B. L. (2004b). Whole proteome prokaryote phylogeny without sequence alignment: A K-String composition approach. *Journal of Molecular Evolution*, **58**(1), 1–11.

Rinke, G., Schwientek, P., Sczyrba, A., Ivanova, N. N., Anderson, I. J., Cheng, J.-F., Darling, A., Malfatti, S., Swan, B. K., Gies, E. A., Dodsworth, J. A., P., H. B., Tsiamis, G., Sievert, S. M., Liu, W.-T., Eisen, J. A., Hallam, S. J., Kyrpides, N. C., Stepanauskas, R., Rubin, E. M., Hugenholtz, P., and Woyke, T. (2013). Insights into the phylogeny and coding potential of microbial dark matter. *Nature*, **499(7459)**, 431–437.

Saitou, N. and Nei, M. (1987). The neighbor-joining method: a new method for reconstructing phylogenetic trees. *Mol Biol Evol*, **4**(4), 406–425.

Sun, J. D., Xu, Z., and Hao, B. L. (2010). Whole-genome based Archaea phylogeny and taxonomy: a composition vector approach. *Chinese Science Bulletin*, **55**(24), 2323–2328.

Tang, F. F., Huang, Y. T., Chang, H. L., and Wong, K. C. (1957). Isolation of trachoma virus in chicken embryo. *Journal of Hyg Epidemiol Microbiol Immunology*, **1**, 109–120.

Wang, H., Xu, Z., Gao, L., and L., H. B. (2009). A fungal phylogeny based on 82 complete genomes using the composition vector method. *BMC Evolutionary Biology*, **9**, 195.

Xu, Z. and Hao, B. L. (2009). CVTree update: a newly designed phylogenetic study platform using composition vectors and whole genomes. *Nucleic Acids Research*, **37**, W174–W178.

Yu, Z. G., Zhou, L. Q., Anh, V. V., Chu, K. H., Long, S. C., and Deng, J. Q. (2005). Phylogeny of prokaryotes and chloroplasts revealed by a simple composition approach on all protein sequences from complete genomes without sequence alignment. *Journal of Molecular Evolution*, **60**(4), 538–45. PMID: 15883888.

Zuo, G. H. and Hao, B. L. (2015). CVTree3 web server for whole-genome-based and alignment-free prokaryotic phylogeny and taxonomy. *Genomic Proteomics and Bioinformatics*. being submitted.

Zuo, G. H., Xu, Z., Yu, H. J., and Hao, B. L. (2010). Jackknife and bootstrap tests of the composition vector trees. *Genomics, Proteomics and Bioinformatics*, **8**, 262–267.

Zuo, G. H., Xu, Z., and Hao, B. L. (2013). Shigella strains are not clones of Escherichia coli but sister species in the genus Escherichia. *Genomics Proteomics Bioinformatics*, **11**, 61–65.

Zuo, G. H., Li, Q., and Hao, B. L. (2014). On K-tuple length in composition vector phylogeny of prokaryotes. *Computational Biology and Chemistry*, **53**, 166–173.

Zuo, G. H., Xu, Z., and Hao, B. L. (2015). Phylogeny and taxonomy of Archaea: a comparison of the whole-genome-based CVTree approach with 16S rRNA sequence analysis. *Life*, **5**, 949–968.

Zuo, G. H., Hao, B. L., and Staley, J. T. (2104). Geographic divergence of "Sulfolobus islandicus" strains by genomic analyses including electronic DNA hybridizztion confirms they are geovars. *Antonie van Leeuwenhock J. Microbiol.*, **105**, 431–435. doi: 10.1007/s10482-013-0081-4.
